# Potential Application of Electrical Stimulation in Stem Cell-Based Treatment against Hearing Loss

**DOI:** 10.1155/2018/9506387

**Published:** 2018-05-08

**Authors:** Mingliang Tang, Xiaoqian Yan, Qilin Tang, Rongrong Guo, Peng Da, Dan Li

**Affiliations:** ^1^Key Laboratory for Developmental Genes and Human Disease, Ministry of Education, Institute of Life Sciences, Southeast University, Nanjing 210096, China; ^2^Co-Innovation Center of Neuroregeneration, Nantong University, Nantong 226001, China; ^3^Joint Research Institute of Southeast University and Monash University, Suzhou 215123, China; ^4^The First Clinical Medical School, Anhui Medical University, 81 Meishan Road, Hefei 230032, China; ^5^Department of Otolaryngology-Head and Neck Surgery, Affiliated Hospital of Nantong University, Nantong 226001, China

## Abstract

Deafness is a common human disease, which is mainly caused by irreversible damage to hair cells and spiral ganglion neurons (SGNs) in the mammalian cochlea. At present, replacement of damaged or missing hair cells and SGNs by stem cell transplantation therapy is an effective treatment. However, the survival rate of stem cell transplantation is low, with uncontrollable differentiation hindering its application. Most researchers have focused on biochemical factors to regulate the growth and differentiation of stem cells, whereas little study has been performed using physical factors. This review intends to illustrate the current problems in stem cell-based treatment against deafness and to introduce electric field stimulation as a physical factor to regulate stem cell behavior and facilitate stem cell therapy to treat hearing loss in the future.

## 1. Introduction

In recent years, with noise, virus infection, ototoxic drug abuse, environmental pollution, and the development and exacerbation of other adverse factors, incidence rates of deafness and hearing loss have gradually increased among the aging population. According to data from the World Health Organization, hearing loss seriously affects the quality of life among 360 million people worldwide, making it a global health problem that cannot be ignored [1]. In general, accumulation of a variety of physicochemical or pathological factors, such as noise and drugs, could ultimately lead to irreversible damage or loss of human inner ear hair cells and/or spiral neuron cells. Therefore, promoting regeneration of hair cells and spiral neurons in order to repair the structure and function of the cochlea has been considered as the best treatment approach. As mammalian hair cells and spiral neurons are not self-regenerative, regenerating damaged cochlear hair cells and spiral neurons, from differentiation of stem cells or progenitor cells, has attracted major research interest in recent years. It was found that supporting cells are a candidate progenitor to replace hair cells in avian cochlea [2]. Since then, there have been increasing investigations conducted on the regeneration of hair cells and spiral neurons in the mammalian inner ear, with the aim to identify intrinsic molecular mechanisms underlying stem cell transplantation, in order to provide a viable clinical approach to treat hearing loss. Many studies have supported the potentials of hearing loss treatment using stem cell transplantation, beginning with a pioneering study by Ito et al. [3]. Furthermore, numerous laboratories have tried to transplant different types of stem cells into the inner ear [4–7]. For example, the bone marrow-derived mesenchymal stem cells were successfully transplanted into the mouse cochlea and were further differentiated into fibrocyte-like cells [8].

## 2. Hair Cell and Spiral Ganglion Neuron (SGN) Regeneration Research

Studies have shown that a type of Lgr5-positive cells in the mouse cochlea has the potential to differentiate into hair cells and is therefore considered as potential cochlear stem cells [9, 10]. Many researchers attempted to study the involvement of Wnt and Notch signaling pathways in promoting the proliferation and differentiation of Lgr5-positive cells for hair cell regeneration. Some researchers have focused on growth factors and proteins of signaling pathways necessary for hair cell regeneration and found that insulin-like growth factor 1 could promote synthesis of DNA in chickens [11]. Li et al. have demonstrated that Notch signaling promoted Lgr5-positive progenitor cells to mitotically generate new hair cells and inhibition of Notch activated the canonical Wnt signaling pathway [12]. Both behavioral and physiological studies have shown that hair cell regeneration is able to restore responsive property and vestibular reflex in the vestibular afferent nerve fibers [11, 13, 14]. With advances in mechanistic research in hair cell regeneration, it is increasingly promising to regenerate hair cells from stem cells in the future.

Noise, brain trauma, and a variety of other diseases can cause damage to cochlear spiral ganglion neurons (SGNs), leading to hearing loss. In the mouse model, acute noise-induced damage to SGNs of peripheral nerve endings resulted in loss of hearing [15]. There is an urgent need to repair SGN damage-induced hearing loss. One new therapy is to induce other types of stem cells to differentiate into neurons to replace the damaged SGNs. In this context, there is also evidence indicating that adult mammalian auditory neurons contain neural precursor cells. Rask-Andersen et al. isolated nestin-positive neural stem cells (NSCs) from adult guinea pig helical ganglia [16]. Although adult mammalian cochlear spiral ganglions have regenerative potential, there have been few observations of their regeneration after injury. In addition, although there are indications that human spiral neurons have a slight regenerative capacity, it has no clinical significance [17]. In recent years, NSC transplantation has become a novel approach in the treatment of neurodegenerative diseases including sensorineural deafness [18]. In the past decade, significant progress has been achieved in stem cell replacement therapy using SGNs to treat hearing loss [19, 20]. With the abovementioned profess, increasing studies have been committed to SGN regeneration using stem cell therapy to treat hearing loss.

However, the inability to control the differentiation of transplanted cells *in vivo* has become a serious problem in the treatment against hearing loss. For example, to selectively regenerate hair cells from the inner ear precursors or spiral neurons from the NSCs is still hard to achieve.

## 3. Stem Cell Transplantation for the Treatment of Hearing Loss

Stem cells, such as adult and embryonic stem cells, are a group of cells with the potential of self-renewal and differentiation [21]. Stem cells have been employed in various fields including tissue engineering [22], regenerative medicine [23], cancer research [24], and various neurological diseases [25]. In particular, Chai et al. have discovered a type of Lgr5-positive cells in the mouse cochlea [9, 10], which could be derived into hair cells and is therefore considered as cochlear stem cells.

Meanwhile, NSCs have been widely used in the treatment of neurodegenerative diseases. It is still challenging to promote the regeneration of neurons from NSCs as well as the functional maturation of newly formed neurons. A stem cell-based approach has been proposed to replace degenerated spiral neurons [26]. Investigations have suggested that differentiation of NSCs into spiral neurons is a viable approach for repairing spiral neuronal damage in the inner ear. NSCs have been reported to have the ability to self-renew and are able to differentiate into neurons, astrocytes, oligodendrocytes, and other major neural tissues. NSCs may differentiate into functional auditory neurons [27]. Due to the self-renewal, pluripotency, migration, good histocompatibility, and low immunogenicity of NSCs, they can be used as excellent seed cells to replace lost spiral neurons to promote their regeneration. For example, studies have demonstrated that adult human mesenchymal-like stem cells isolated from nasal tissues could be employed to restore lost SGCs in cochlear explant culture isolated from neonatal rats following challenge [28].

## 4. Electric Field (EF) Stimulation Can Regulate Stem Cell Behavior

There is a complex in vivo interaction between stem cells and their surrounding environment, called the niche, which involves biochemical factors, extracellular matrix components, physical factors, and cell-cell interactions [29]. Stem cell niche determines the fate of stem cells; therefore, when the stem cells are studied in vitro, spatial structure of the niche changes, resulting in new obstacles in research. Finding the right factors to regulate the fate of stem cells will greatly improve the development of stem cell therapy. In this context, electric field (EF) stimulation is a common strategy of using physical stimulation to regulate cell behavior both *in vivo* and *in vitro*.

EF stimulation is one of the important guidance cues regulating signaling pathways to induce cellular events such as proliferation and migration in pathological and physiological processes, such as tissue regeneration, embryonic development, and wound healing [11, 30–33]. To date, research on the regulation of cell microenvironment by EF stimulation has mainly focused on using EF to excite receptors and ion channels on the cell membrane to drive depolarization, hyperpolarization, proliferation, migration, and differentiation [34, 35].

Over the past few decades, researches have shifted from using endogenous EFs to promote wound healing to using artificial EF stimulation to excite nerves to induce muscle contraction [36]. Endogenous EFs are known to influence cell migration in vivo. For instance, physiological levels of electrical stimulation applied on OPCs isolated from neonatal Sprague-Dawley rats could affect the in vitro migration of OPCs via *β*1 integrin [37]. However, the cathodal or anodal electrotaxis is cell-type dependent and most of the cell types are recruited to the cathodal pole of the EF [38–43]. It was reported that neural stem/progenitor cells under physiological EF strength migrated towards the cathode at an increased rate [44]. EF stimulation not only has a significant effect on the migration of stem cells but also has an irreversible effect on their differentiation. A recent study has shown that integrating the conducting carbon nanofibrous scaffold with electrical stimulation enhances NSC functions [45]. These findings indicate that electrical stimulation is indeed an effective physical method to regulate physiological cellular behavior. In a natural situation, the occurrence of an endogenous EF (10–1800 mV/mm) is a prerequisite for normal neuron development in frogs and chick embryos [46]. Direct EFs play a crucial role throughout the development of the nervous system [31, 46]. EFs have profound effects on nerve growth, guidance, and branching during neural construction, where an EF as low as 10 mV/mm was able to frequently turn the growth cones towards the cathode [46]. In the same study, EF was also demonstrated to play a critical role under pathological conditions, where EFs were observed in damaged axons to regenerate axons [46]. In addition, small EFs applied on animal models of spinal cord injury resulted in functional improvements in these central nervous system injury models [47]. Electrical stimulation was also reported to promote NSC differentiation towards nerve regeneration to improve neural circuit reconstruction [48–50].

## 5. Perspectives

Cochlear implant has become one of the most successful functional artificial organs in modern medicine. It is an electronic device that restores or obtains hearing in individuals with severe hearing loss and even complete deafness. The device converts sound waves into electric signals that directly stimulate spiral nerve cells and auditory nerve fibers, independent of hair cells. With continuous research efforts, cochlear implants have been widely used clinically to treat hearing loss. As the only medical device capable of restoring hearing and speech abilities among deaf patients, cochlear implant has been widely applied since its FDA approval in the mid-1980s. By 2015, over 300,000 patients worldwide have received cochlear implants, with this figure increasing at the rate of tens of thousands every year [51]. In 1995, China introduced multiguided cochlear implant technology, which has been popularized throughout the country and benefited over 100,000 patients.

As mentioned above, stem cell-based therapy has exhibited promising potential for hearing loss treatment. However, it is still a big challenge to construct a more physiologically relevant microenvironment to facilitate basic research and clinical application using different types of materials. The cellular microenvironment directly affects the growth trend of cells and even determines their fates; therefore, constructing a suitable microenvironment for cell growth is crucial for the successful transplantation of stem cells. Stem cells often exhibit different characteristics when they are transplanted into recipients and the traditional culture systems are two dimensional, using multiwell plates, coverslips, and petri dishes [52, 53]. Due to the lack of tissue-specific architecture, mechanical and biochemical cues, and cellular communication in artificial environment, although these traditional two-dimensional cell culture systems are valuable for basic research [54], they are unsuitable for clinical studies demanding a large number of cells for transplantation and regeneration [55]. Therefore, it is crucial to build a three-dimensional (3D) stem cell culture system that mimics the in vivo stem cell microenvironment. 3D culture systems not only preserve the native extracellular matrix structure but also more accurately represent the physiological microenvironment [56]. Due to these excellent features, 3D systems have been widely used in stem cell culturing. Currently, there are two major types of 3D scaffold materials: natural and artificial. Graphene has unique physicochemical properties, such as high specific surface area, high charge mobility, and good mechanical strength, and therefore has been widely used in drug transport [57], stem cell engineering [58], and oncology. For example, human mesenchymal-like stem cells grown on 3D graphene foam scaffold exhibited enhanced differentiation into osteogenic lineages [59]. As an excellent conductive material, graphene has been used as a good neural interface material, which significantly promoted the differentiation of NSCs into neurons [58]. Nevertheless, there are still some limitations to the use of these scaffolds. With the addition of EF stimulation to regulate cell proliferation, differentiation, and migration, combined cochlear and stem cell transplantation may become a new strategy for the treatment against hearing loss.

## 6. Challenge

It has been reported that electrical stimulation can regulate the differentiation of stem cells, in which special biological materials play an important role [60]. These discoveries have opened a new opportunity for the combined treatment against hearing loss using cochlear implant and stem cells. However, there are still many problems that need to be solved to better implement the treatment against hearing loss with stem cell transplantation and cochlear implant. For example, the strength of electrical stimulation has a crucial effect on the behavior of stem cells. Different intensities of EF stimulation induce differentiation of stem cells into neurons and neuron maturation to different extents [61]. On the other hand, the complex interactions between the substrate materials and the growing cells also influence the behavior of the cultured stem cells. Finding suitable materials, which are biocompatible and conductive, could greatly facilitate stem cell transplantation, meanwhile incorporating electrical stimulation as a regulatory factor. For example, graphene, a new nanomaterial with specific physicochemical properties, has been reported to be a suitable stem cell scaffold that could deliver electrical stimulation and significantly promote NSC differentiation into neurons [58]. Furthermore, specific molecular events underlying the EF regulation on stem cell behavior are still largely unknown. Further studies are warranted to advance the understanding and treatment on hearing loss using stem cell therapy.

## 7. Conclusion

Stem cell transplantation technology has greatly benefited deaf patients. On the other hand, there is an urgent need for new physical methods other than biochemistry to solve potential problems in stem cell transplant therapy. EF stimulation is a common strategy of using physical stimulation to regulate cell behavior both *in vivo* and *in vitro*. Prominent research progress has been achieved using electrical stimulation to regulate stem cell behavior. It would be revolutionary to combine electrical stimulation and stem cell transplantation with other biomaterials in the future to improve the treatment of deafness.
